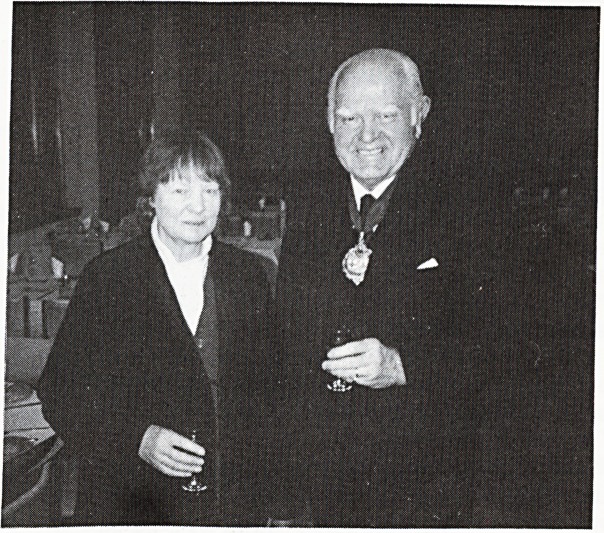# An Evening with Iris Murdoch

**Published:** 1986-08

**Authors:** M. G. Wilson


### An evening with Iris Murdoch

**Meeting of the Bristol Medico-Chirurgical Society on March 12th, 1986.**

Our President, Dr Joze Jancar invited his old friend, the novelist Iris Murdoch to come and speak to us, she agreed, but said that rather than deliver a formal speech she would like to have a discussion on subjects brought up by members of the Society. He told us how their friendship began in 1948 when Joze Jancar was a medical student in a refugee camp in Graz and Iris Murdoch was Deputy Director of UNRRA (United Nations Relief and Rehabilitation Agency) in the area (Miss Murdoch modestly revealed that the UNRRA team consisted of the Director and herself).

The discussion was initiated by a panel consisting of Professor Russell Davies, Mr John Crossley and Dr Terence Steen. Professor Davies began by quoting from something Miss Murdoch had written years ago linking the novelists function with therapy. While perhaps art might at times have a therapeutic effect, she did not think that should be the aim of the artist. Art should seek something fundamental – whatever the artist chooses. Here your correspondent feels that his notes and his memory are not adequate to recall or do justice to the discussion. In fact he can do no better than record in random fashion, a few of the thoughts and ideas which the questions evoked and which he managed to capture, eg. 'We live in a post-Freud and post-Marx era since when attitudes have changed fundamentally ... the hours and hours of talking to a psychoanalyst might be just as therapeutic if they had been spent with a friend ... Freud got his idea of the subconscious from Plato ... Pornography is the end-point of the degradation of art ... art which is the opium of the people is bad art, made in totalitarian states to serve the ends of the state ... where the good citizen is shown as noble and successful'.

Mr Crossley asked Miss Murdoch which novelists of the past 50 years she thought had been most significant. Miss Murdoch began by saying that she was not a reader of the contemporary novel and could not speak about recent work '... 'Joyce disturbed the world of the novel with Ulysses, but he was not a good model, rather a dead-end, he changed the use of language, he was cold, sending out rays of power ... Kafka – metaphysical and deep, hard to imitate ... Virginia Woolf changed the tone of the novel – after her things became impossible which before had been accepted, e.g. the narrative style of Dickens or Thackeray, she admired her but did not like her ... D. H. Lawrence was a great writer, but not as great as often thought – he intrudes himself too much ... Hardy was a more erotic writer than Lawrence ... Proust was the greatest of the French novelists ... J. C. Powys, if not a great writer, was one whose work she admired ... Shakespeare is the greatest writer of all time, his range and scope is astonishing, his character is unknown, except perhaps through his sonnets, and this adds to his fascination, what a disaster it would be if a contemporary biography of him was unearthed'.

Dr Steen asked her to look into the future and tell us whether she thought the social fabric was collapsing. She did not think so. 'The future will see this age as being catastrophically revolutionary ... the prophets are usually wrong – as was Marx ... Science shows us that the cosmos is a language of its own...there may become two sorts of people, those who can speak computer language and those who don't and they will read novels'.

The opportunity to ask questions was then put to the floor of the house and the discussion continued –' Why were all the different characters in her books linked to each other? Are philosophy and psychology linked? Is magic escapism? How does your inspiration come to you? Why are there so many successful women writers and so few composers?'. Amongst the replies ... ' Art is funny, full of tricks and jokes ... never laugh in public in a totalitarian state ... to start writing a book you look out of the window thinking about the initial plan and wait till the live ideas appear'.

These random notes can give little idea of the lucid and ordered stream of ideas that flowed in response to each question which was like throwing a pebble into a calm pool with deep undercurrents and watching an intricate and unpredictable pattern of ripples.

We had a very memorable evening. It was a great privelege to have this glimpse into the mind of one of the most original and individual writers of our time. My only regret is that nobody thought to record this wonderful soiree on tape.

M. G. Wilson

## Figures and Tables

**Figure f1:**